# Inbreeding and adaptive plasticity: an experimental analysis on predator-induced responses in the water flea *Daphnia*

**DOI:** 10.1002/ece3.1545

**Published:** 2015-06-19

**Authors:** Ine Swillen, Joost Vanoverbeke, Luc De Meester

**Affiliations:** Laboratory of Aquatic Ecology, Evolution and Conservation, University of LeuvenCharles Deberiotstraat 32, 3000, Leuven, Belgium

**Keywords:** *Daphnia*, inbreeding, plasticity, predator stress

## Abstract

Several studies have emphasized that inbreeding depression (ID) is enhanced under stressful conditions. Additionally, one might imagine a loss of adaptively plastic responses which may further contribute to a reduction in fitness under environmental stress. Here, we quantified ID in inbred families of the cyclical parthenogen *Daphnia magna* in the absence and presence of fish predation risk. We test whether predator stress affects the degree of ID and if inbred families have a reduced capacity to respond to predator stress by adaptive phenotypic plasticity. We obtained two inbred families through clonal selfing within clones isolated from a fish pond. After mild purging under standardized conditions, we compared life history traits and adaptive plasticity between inbred and outbred lineages (directly hatched from the natural dormant egg bank of the same pond). Initial purging of lineages under standardized conditions differed among inbred families and exceeded that in outbreds. The least purged inbred family exhibited strong ID for most life history traits. Predator-induced stress hardly affected the severity of ID, but the degree to which the capacity for adaptive phenotypic plasticity was retained varied strongly among the inbred families. The least purged family overall lacked the capacity for adaptive phenotypic plasticity, whereas the family that suffered only mild purging exhibited a potential for adaptive plasticity that was comparable to the outbred population. We thus found that inbred offspring may retain the capacity to respond to the presence of fish by adaptive phenotypic plasticity, but this strongly depends on the parental clone engaging in selfing.

## Introduction

Inbreeding and inbreeding depression, the reduced fitness of inbred individuals, is an intensively studied field in evolutionary biology (Charlesworth and Charlesworth 1987; Saccheri et al. 1998; Keller and Waller 2002; Bakker et al. 2010; Bijlsma and Loeschke 2011; Fox et al. 2011; Reed et al. 2012). Numerous studies report inbreeding in a large variety of taxa, ranging from invertebrates to mammals (e.g., Keller et al. 1994; Madsen et al. 1996; Wade et al. 1996; Saccheri et al. 1998; Ross-Gillespie et al. 2007; Thunken et al. 2007; Jamieson 2011). In these studies, the degree of inbreeding depression varies widely, with some populations showing minor or no effects of inbreeding, while in others inbreeding depression is strong (Keller and Waller 2002). While inbreeding depression is often pronounced, purging, that is, the loss of genetic load from a population through selective removal of individuals expressing deleterious alleles, may lead to a quick fitness rebound in the inbreeding population (Crnokrak and Barrett 2002), thereby strongly reducing the costs of inbreeding over time.

Inbreeding depression is dependent on the environment, and several studies have emphasized that inbreeding depression is enhanced under stressful environmental conditions (Armbruster and Reed 2005; Fox and Reed 2011). Quantifying the degree of inbreeding depression under stressful conditions is highly relevant in the context of the conservation of small populations that are exposed to environmental stressors (Hedrick and Kalinowski 2000; Armbruster and Reed 2005). An important mechanism for populations to survive in variable and potentially stressful environmental conditions is phenotypic plasticity, that is, a change in phenotype for a given genotype as a function of the environment (Schlichting and Pigliucci 1998; Ghalambor et al. 2007; Lande 2009; Bateson et al. 2011). Inbreeding may cause a loss of adaptive plasticity responses, contributing to reduced fitness under environmental change and further increasing inbreeding depression. The reason inbreeding is likely to affect the capacity to be plastic in organisms is threefold: (1) increased homozygosity in inbreds may decrease the diversity of “plasticity genes” that underlie phenotypic plasticity (Via et al. 1995), leading to less opportunities to express phenotypic plasticity. (2) if phenotypic plasticity is the result of “allelic sensitivity,” where a particular allele has varying effects on the phenotype depending on the environment (Via et al. 1995), we would expect inbreeding to less strongly impact the capacity of individuals to show adaptive phenotypic plasticity. (3) phenotypic plasticity may be associated with costs (e.g., DeWitt 1998; DeWitt et al. 1998; Riessen 1999; van Kleunen et al. 2000), so that reduced energy levels in inbred individuals may render them less capable to develop and maintain plastic responses. The few studies that have investigated effects of inbreeding on the capacity to show plastic responses to stressors show either strong (Auld and Relyea 2010) or no effects of inbreeding on adaptive plasticity (Kristensen et al. 2011; Luquet et al. 2011).

Here, we use the water flea *Daphnia magna* to quantify inbreeding depression with respect to phenotypic plasticity upon fish predation pressure. Fish predation is a key structuring factor in zooplankton communities (Kerfoot and Sih 1987) and *Daphnia* populations (Cousyn et al. 2001). Fish predation risk is also a stress factor (Pauwels et al. 2005, 2010) and is a suitable stressor to quantify adaptive phenotypic plasticity as we have good predictions on what the adaptive phenotypic plasticity responses to visual predators are (e.g., smaller size at maturity and increased energy allocation to reproduction; Dodson 1989; De Meester and Cousyn 1997; Spaak and Boersma 1997; Weber and Declerck 1997;Boersma et al. 1998; Tollrian and Harvell 1999; Bourdeau et al. 2013; Carter et al. 2013). The water flea *Daphnia magna* has a cyclical parthenogenetic life cycle, alternating a varying number of generations of asexual reproduction with regular (often yearly) bouts of sexual reproduction, the latter typically triggered by unfavorable environmental conditions (Miner et al. 2012). Genotypic diversity in *Daphnia* populations can be low for two reasons. First, upon colonization of a new patch by only a few resting eggs, clonal reproduction can quickly lead to a numerically large but genotypically small population, potentially leading to long-lasting founder effects (Boileau et al. 1992; De Meester et al. 2002). Second, in a well-established population in which the growing season starts with the hatching of a large number of clones from the dormant egg bank, clonal selection during the course of the season can strongly reduce clonal diversity by the end of the growing season (Vanoverbeke and De Meester 2010). As a result, the number of genotypes engaging in sexual reproduction may be low. This low genetic diversity at the end of the growing season entails the risk that sexual offspring will be inbred. Additionally, mutations that have accumulated during long periods of asexual reproduction, may be exposed after sexual reproduction, potentially adding to a population decrease in fitness (Caceres et al. 2009). Several studies have quantified inbreeding depression in laboratory and wild populations of *D. magna* (e.g., De Meester 1993; Ebert et al. 2002; Haag et al. 2002), and inbreeding depression was in most cases found to be severe. However, no studies on inbreeding depression in *Daphnia* have yet looked at the consequences of inbreeding for adaptive phenotypic plasticity, despite the fact that *Daphnia* clones generally exhibit strong phenotypic plasticity with respect to antipredator defenses (e.g., Dodson 1989; De Meester and Cousyn 1997; Spaak and Boersma 1997; Weber and Declerck 1997; Boersma et al. 1998; Bourdeau et al. 2013; Carter et al. 2013).

The aim of this study was threefold. First, we quantify whether inbreeding depression is still detectable after an initial phase of mild purging under benign laboratory conditions (hypothesis 1). Under inbreeding depression, we expect higher mortality, later maturation, smaller clutch sizes, and overall lower reproductive output in inbreds as compared to outbreds. Second, we assess whether inbreeding depression is stronger in the presence than in the absence of a biotic stressor, predation risk by fish (hypothesis 2). Third, we asked whether inbred lineages retain the capacity to show adaptive phenotypic plasticity upon exposure to fish kairomones (hypothesis 3). If lineages are capable of adaptive phenotypic plasticity under fish predation stress, we expect earlier maturation (as this decreases the probability of being preyed upon before reproduction), higher reproductive output (as larger clutches contain smaller offspring, and being small is beneficial in the presence of visual predators, and as larger clutches may provide the maternal clone with at least some successful offspring before she (or a number of the offspring) are preyed upon), and decreased size at maturity (as fish are visual predators) upon exposure to fish kairomones (Weider and Pijanowska 1993; Boersma et al. 1999).

## Materials and Methods

### Generating inbred families and the outbred subpopulation

Selfed offspring families were obtained by stimulating the production of sexual eggs in monoclonal populations of two maternal clones (I2 and I3) that were hatched from dormant eggs collected in Langerodevijver (50°49′42.20″N – 4°38′23.69″E), a 17 ha fish pond near Leuven, in the center of Belgium. We successfully induced sexual reproduction by culturing *Daphnia magna* in 1 L jars under varying photoperiod and without controlling population densities, leading to food shortage (Alekseev and Lampert 2001). Cultures were kept at 20°C in aged tap water. Jars were cleaned, and half of the medium refreshed twice a week. Cultures were fed 5 × 10^5^ cells/mL of the green alga *Scenedesmus obliquus* daily. The light regime was switched between a long-day photoperiod (16L:8D) during 5 days alternated with a 2-day short-day photoperiod (8L:16D). Crowding combined with changes in photoperiod is known to induce sexual reproduction in *D. magna* (De Meester and De Jager 1993). The dormant eggs that were produced in these inbreeding cultures were removed from all jars twice weekly and stored in eppendorf tubes in the dark at 4°C for several weeks before exposing them to hatching conditions. No hatching of dormant eggs occurred in the cultures as all ephippia were removed shortly after release and a period dominated by adverse conditions such as cold or drought are needed to break diapause of dormant eggs of *D. magna* (De Meester and De Jager 1993). For the outbred population, we used clonal lineages that were derived from the dormant egg bank of Langerodevijver by collecting dormant eggs in the field and subsequently hatching these dormant eggs in the laboratory. These hatchlings are representative of the genetic variation residing in the natural dormant egg bank. The average inbreeding coefficient F_is_ in the outbred population, as measured across twelve microsatellite markers, was 0.16 (as compared to 0.5 in the inbred families we used) and we found significant deviations from expected heterozygosity for 2 of 12 microsatellite markers only (processed sample size = 100 individuals).

### Early extinction of lineages under benign conditions

Starting from 100 to 294 lineages of each subpopulation (i.e., two inbred families and one outbred population), we recorded loss of clones during the first 8 weeks after hatching (5–6 clonal generations) due to inviability or sterility. During these first 8 weeks, cultures were cleaned twice per week and fed daily with the green algae *S. obliquus* (1 × 10^5^ cells/mL). By doing so, we were able to record “purging” of genotypes that suffer from inbreeding depression to such an extent that they are not able to establish monoclonal populations under relatively benign conditions. We assume that the loss of these lineages was not due to strong selection but because of these lineages suffered from severe inbreeding depression caused by homozygosity of strongly deleterious or (sub)lethal, alleles (causing death or sterility). During these first 8 weeks after hatching, we lost 7% of the clones of inbred subpopulation I2 (10 of 142), 56% of the clones of inbred subpopulation I3 (195 of 294) and 3% (3 of 100) of the outbred subpopulation. After these 8 weeks, all lineages were kept for several additional months in culture under standardized stock conditions in the laboratory (20°C, 16L:8D photoperiod, aged tap water as medium, fed 1 × 10^5^ cells/mL of the green alga *S. obliquus* twice weekly, no control for densities) before being involved in experiments. Additional losses during this period were very low (<5%).

### Life table experiment

Using our isolated inbred and outbred clonal lineages, we carried out a life table experiment to quantify life history trait values up to the release of the second clutch. To minimize interference from maternal effects, we cultured all clones under standardized culturing conditions (16:8 h L:D cycle, 24 h aerated tap water, cleaned three times per week, and daily fed 1.2 × 10^5^ cells/mL of the green algae *S. obliquus*) in the absence of fish for two generations prior to the experiment. To start up each new generation, including the experimental generation, we used 24-h old juveniles from the second clutch of the previous generation. The experiment was run in a full factorial design with 8–12 clones per subpopulation (i.e., two inbred families and one outbred group of clones), two treatments (absence and presence of fish kairomones), and three replicate individuals per clone (in total, we had 263 experimental units). Individuals were cultured separately in 210-mL jars in a climate-controlled room (20°C) with long-day photoperiod (16:8 L:D). Individuals subjected to the fish kairomone treatment were cultured in ¾ aged tap water and ¼ fish-conditioned medium, which was obtained by filtering 30 L of water in which three golden ides (*Leuciscus idus melanotus*) had swum for 24 h over a 125 *μ*m mesh-sized sieve. Fish were fed in separate aquaria to avoid the presence of *Daphnia* alarm cues in the fish medium. This fish kairomone treatment mimics high densities of fish (Cousyn et al. 2001). Individuals in the nonfish treatment were cultured in aged (24 h, aerated) tap water. All jars were cleaned and medium refreshed daily, and animals were daily fed 1.2 × 10^5^ cells/mL of the green algae *S. obliquus*. During the course of the experiment, we recorded mortality and the following life history traits for surviving individuals: age and body size at maturity, size of the tail spine at maturity, total number of juveniles (which is maximally the sum of the number of juveniles in the first and second clutch as the experiment was terminated after release of second clutch), and body size of juveniles in first clutch. The experiment was terminated individually after the release of second clutch. Size measurements were taken with a stereomicroscope at a magnification of 20× for juveniles and 40× for adults. Body size was measured from the top of the eye down to the base of the tail spine (i.e., excluding the tail spine). Tail spine size was measured from the base to the tip of the tail spine. Performance “*r*” was calculated iteratively for each individual (including reproducing animals only) based on the timing of reproduction and the number of offspring, following the Euler equation (∑ *e*^−*rx*^ *l*_*x*_ *m*_*x*_ = 1; Roff 1997). We call this variable “performance” (Van Doorslaer et al. 2009) rather than “population growth rate,” as we ignore mortality (*l*_*x*_ = 1).

### Statistics

To compare life history traits between the different subpopulations (i.e., two inbred families and the outbreds) in the absence and presence of fish kairomones, we performed a general(ized) linear mixed model for each trait. As categorical factors in our models, we included “Clonal line” as a random factor nested in “Subpopulation,” and “Subpopulation,” “Treatment” (fish kairomones absent or present) and the “Subpopulation × Treatment” interaction as fixed factors.

A significant “Subpopulation” main effect indicates the possible presence of ID. We checked for the effective presence of inbreeding depression by inspection of the graphs along with performing Tukey–Kramer (TK)-adjusted post hoc LS means comparison test in which we looked at significant differences between mean trait values of each inbred subpopulation (separately) and the outbreds (*P*-values of these differences are summarized in Table2).

A significant “Treatment” main effect indicates the possible presence of adaptive plasticity. We checked for the effective presence of adaptive phenotypic plasticity by inspection of the graphs along with performing TK post hoc LS means comparison tests in which we looked at significant differences in mean trait values between the treatments with and without fish kairomones within a single subpopulation (*P*-values of these differences summarized in Table2).

A significant “Subpopulation × Treatment” interaction effect indicates the possible aggravation of ID by a stressor, as well as an effect of inbreeding depression on plasticity.

For all traits except mortality, we ran a generalized linear mixed model. To conform to assumptions, age at maturity was log-transformed before analysis. Size of the tail spine was expressed as the percentage of total body size at maturity. To analyze mortality, we ran a generalized linear mixed model with binomial error distribution and logit link function, in which the response variable was the number of dead individuals per clonal line as compared to the total number of experimental individuals per clonal line. All analyses were performed in SAS 9.3 (SAS Institute Inc., -2010^2002^).

## Results

### Hypothesis 1: Is inbreeding depression still detectable after purging?

We found evidence for inbreeding depression, even after purging, in several life history traits we studied in our life table experiment, indicated by a significant effect of “Subpopulation” for all the traits we studied here.

**Figure 1:**
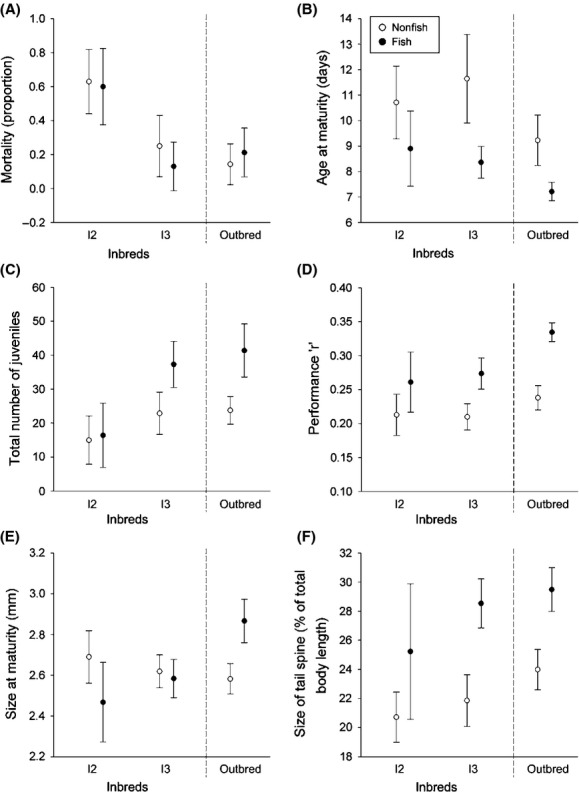
Mortality (A), age at maturity (B), total number of juveniles (C), performance “*r*” (D), size at maturity (E), and size of the tail spine (F) of two inbred subpopulations generated as selfed offspring of clones isolated from Langerodevijver (left and middle panel in each graph) and a group of outbred clones directly hatched from Langerodevijver (LRV) (middle panel of each graph). Life history traits as quantified in a life table experiment in the absence (open symbols) and presence (closed symbols) of fish kairomones. Error bars represent 2× standard error of the mean.

We found that the mildly purged inbred family I2 suffered inbreeding depression for several traits: clones from family I2 overall had higher mortality rates, matured later, and had shorter tail spines than outbreds (Tables1 and 2, Fig.1A–F) irrespective of the condition they were reared in. Moreover, in the presence of fish, I2 clones had a lower total number of juveniles and overall lower performance than outbred clones (Tables1 and 2, Fig.1C and D).

**Table 1:** Results of general(-ized) linear mixed models (for age at maturity, size at maturity, size of tail spine, and performance “*r*”) or generalized linear mixed models (for total number of juveniles and mortality) testing for the effect of subpopulation (“Subpopulation”), exposure to fish kairomones (“Treatment”), and their interaction on life history traits as quantified in a life table experiment using clonal lineages from two inbred families and a group of outbred clones. Significant *P-*values are indicated in bold italics

	Age at maturity					Size at maturity					Total number of juveniles				
	DF	Type III SS	MS	*F*	*P*	DF	Type III SS	MS	*F*	*P*	Num DF	Den DF	*F*	*P*
Clonal line (Subpopulation)	25	0.32	0.01	2.25	***0.003***	25	2.50	0.10	2.28	***0.00***	–	–	–	–
Subpopulation	2	0.14	0.07	12.43	***<0.0001***	2	0.40	0.20	4.60	***0.01***	2	29	6.77	***0.004***
Treatment	1	0.18	0.18	31.67	***<0.0001***	1	0.01	0.01	0.34	0.56	1	127	116.25	***<0.0001***
Subpopulation × Treatment	2	0.01	0.01	1.27	0.287	2	1.24	0.62	14.16	***<0.0001***	2	127	18.74	***<0.0001***
Error	91	0.51				91	3.99							

	Performance “*r*”					Size of tail spine					Mortality				
	DF	Type III SS	MS	*F*	*P*	DF	Type III SS	MS	*F*	*P*	Num DF	Den DF	*F*	*P*
Clonal line (Subpopulation)	25	0.12	0.00	2.62	***0.00***	25	837.97	33.52	2.55	***<0.001***	–	–	–	–
Subpopulation	2	0.06	0.03	16.33	***<0.0001***	2	182.31	91.15	6.93	***0.002***	2	29	6.97	***0.003***
Treatment	1	0.10	0.10	58.01	***<0.0001***	1	580.84	580.84	44.16	***<0.0001***	1	23	0.14	0.713
Subpopulation × Treatment	2	0.01	0.01	3.59	***0.03***	2	27.49	13.74	1.04	0.356	2	23	0.98	0.389
Error	90	0.16				91	1196.85							

**Table 2:** Results of Tukey–Kramer post hoc LS means comparisons in the general(ized) linear models (for age at maturity, size at maturity, size of tail spine, and performance “*r*”) or generalized linear mixed models (for total number of juveniles and mortality). As for mortality, age at maturity and size of the tail spine the “Subpopulation × Treatment” interaction was not significant, we show *P*-values for post hoc LS means comparisons within the “Treatment” (upper left) and “Subpopulation” (upper right) main effects. For total number of juveniles, performance and size at maturity, our general(ized) linear mixed models indicated a significant “Subpopulation × Treatment” interaction. For these traits, *P*-values for all relevant pairwise LS means comparisons are shown here, testing for significant differences (1) between inbred family I2 or I3 and the outbreds, within a rearing condition (bottom left and middle column) or (2) between the no-fish and the fish condition, within a single subpopulation (bottom right column). Significant *P-*values are indicated in bold italics

	Fish versus no-fish	Versus outbreds (overall)	
		I2	I3
Mortality	0.713	***0.005***	0.980
Age at maturity	***<0.0001***	***<0.001***	***<0.001***
Size of tail spine	***<0.0001***	***0.001***	0.562

	Fish versus no-fish			Versus outbreds in absence of fish		Versus outbreds in presence of fish	
	I2	I3	Outbreds	I2	I3	I2	I3
Total number of juveniles	0.999	***<0.0001***	***<0.0001***	***0.047***	1.000	***0.002***	0.999
Performance	0.241	***<0.001***	***<0.0001***	0.269	0.401	***<0.001***	***<0.001***
Size at maturity	0.338	0.982	***<0.0001***	0.545	0.958	***0.001***	***<0.0001***

Inbred family I3, which suffered strong purging, did not show pronounced inbreeding depression. In the absence of fish, inbred clones from family I3 performed equally well as outbreds, except for their delayed maturation as compared to the outbreds (Tables1 and 2, Fig.1B). In the presence of fish, performance of the I3 clones was less than the performance of outbreds (Tables1 and 2, Fig.1D), but no inbreeding depression was present for any of the other traits.

### Hypothesis 2: Is inbreeding depression stronger in the presence of a stressor?

In particular, for clones belonging to family I2, the effects of inbreeding were indeed more pronounced under stress: in the presence of fish, these clones suffered inbreeding depression for all studied traits. Notably, they had fewer offspring and lower performances than outbred clones. For these two traits, family I2 did not suffer inbreeding depression in the absence of fish (Tables1 and 2, Fig.1C and D). For clones belonging to family I3, inbreeding depression was not pronounced at all, and the presence of a stressor only enhanced the difference between inbreds and outbreds for one trait, performance (Table2, Fig.1D).

### Hypothesis 3: Does inbreeding affect the ability to be phenotypically plastic?

Both the inbreds from family I3 and the outbred clones showed a capacity to be phenotypically plastic and alter life history in the presence of fish (Tables1 and 2, Fig.1). Inbred clones from family I3 were therefore able to track the outbred clones and perform almost equally well under both conditions. Clones from inbred family I2 responded less to the presence of fish by changes in their life history: total number of juveniles and performance, the more important fitness-related traits, did not alter between the no-fish and the fish condition in this family (Table2, Fig.1).

## Discussion

### Inbreeding depression after purging

If we combine mortality during purging (i.e., early extinction of lineages under benign conditions prior to experiments) and mortality during the life table experiment, both inbred subpopulations (i.e., families) showed higher total mortalities than the outbred subpopulation. Survival was up to 60% lower in inbred families as compared to the set of outbred clones kept under the same conditions (mortality due to purging: I2: 7% – I3: 56% – outbreds: 3%; mortality during the experiment: I2: 60–62% – I3: 13–25% – outbreds: 14–21%). This indicates strong inbreeding depression and is in line with the results of earlier studies analyzing genetic load in *Daphnia* (Innes 1989; De Meester 1993; Deng and Lynch 1998; Haag et al. 2002). Alternatively, genetic slippage may cause populations to move away from selective optima (Lynch and Deng 1994). However, genetic slippage would most likely have occurred, if at all, to the same extent in all our studied subpopulations and therefore cannot account for the large differences we found here between inbreds and outbreds. In general, we also found evidence of inbreeding depression for several other fitness-related traits in the life table experiment, although the presence of inbreeding depression clearly differed between inbred families. Inbred family I2, which showed only moderate purging, suffered substantial inbreeding depression in our life history experiment: it had higher mortality rates during the experiment, matured significantly later than the outbreds, and had smaller clutch sizes. Family I2 also had a lower performance and had smaller tail spines in the presence of fish compared to the outbreds. The other inbred family, I3 did not suffer strong inbreeding depression and generally matched outbred clones quite well. It seems that while in this family initial mortality was very high (initial loss of clones was up to 56%), it lead to a more effective purging of the genetic load, leading to decreased inbreeding depression in life history traits. So overall, we find that strong purging in our study organism *may* aid in relieving inbreeding depression from a population, but this strongly depends on the family. The effectiveness of purging has been shown to vary strongly across species (reviewed in Crnokrak and Barrett 2002) and can be explained by the fact that the benefits of purging are strongly dependent on the genetic architecture of the inbreeding population. More specifically, purging can only reduce inbreeding depression when the underlying cause for inbreeding depression is the presence of (semi)lethal recessive alleles (i.e., when inbreeding depression is caused by partial dominance). If inbreeding depression is caused by overdominance, purging does not relieve inbreeding depression. Therefore, it may not be surprising that we found such strong differences between both families in the degree of purging and the subsequent occurrence of inbreeding depression, even though we studied only two families here.

### Inbreeding depression under stress

The presence of fish kairomones overall affected several of the traits we measured in our life table experiment. The presence of fish increased the degree of inbreeding depression for both families for one key fitness trait, that is, performance, indicating that the presence of a stressor may have strong fitness consequences for inbreeding families. In family I2, total number of juveniles was affected by the presence of fish in addition to performance, further increasing overall inbreeding depression in this family. We note that for size at maturity, there was also a significant “Subpopulation × Treatment” effect, but the phenotypic changes observed in the outbreds were opposite to the ones predicted under fish predation (see below), and differences between inbreds and outbreds with respect to body size can thus not be interpreted in terms of inbreeding depression. As we only studied two families here, we can only cautiously make a general conclusion about the effects of stress on inbreeding depression: overall, our results do suggest that the presence of a stressor may increase inbreeding depression in *Daphnia magna*, but this strongly depends on the family under study. These results are in line with previous findings regarding the impact of stress on inbreeding depression. The effects of stress on inbred populations have been widely studied in the context of conservation biology, and it is current consensus that stress generally aggravates inbreeding depression (reviewed in Armbruster and Reed 2005; Bijlsma and Loeschke 2011; Fox and Reed 2011; Reed et al. 2012; but see Waller et al. 2008).

### Inbreeding and adaptive phenotypic plasticity

With respect to the ability to show adaptive phenotypic plasticity upon inbreeding, we found that in inbred family I2, which experienced only mild purging, there was no difference between the control treatment and the fish treatment for total number of juveniles, performance, and size at maturity. These results indicate that clones from this family are hardly capable of altering their life history to better suit environmental conditions. In contrast, for inbred family I3, which suffered strong initial purging, we found that inbred clones generally retained the capacity to show adaptive phenotypic plasticity in response to fish kairomones. Clones from this family exhibited levels of plasticity similar to the outbreds: they matured earlier (average of ±4 days) and showed a higher total number of juveniles and higher performance in the presence of fish kairomones, which is in line with a multitude of studies on predator-induced changes in life history traits in *Daphnia* (Weider and Pijanowska 1993; Reede 1995; Boersma et al. 1999). Our observation that the animals in the outbred subpopulation were larger at maturity in the presence than in the absence of fish is unexpected, as fish are visual predators and being larger thus increases predation risk. This result is in contrast with the literature (Weider and Pijanowska 1993; Boersma et al. 1998, 1999) and is difficult to explain. The animals did detect the kairomones, as size at maturity did change in the presence of fish kairomones and we did observe adaptive responses to the presence of kairomones for other life history traits. During a field monitoring study in Langerode- vijver, we observed that the *D. magna* population reaches high densities during the spring until the entire population is very rapidly, even within a few days, eaten by fish (Vanhamel et al., I. Swillen, pers. obs.), presumably when the young-of-the-year fish reach the size that they move outside the vegetation and prey massively on *D. magna*. Although speculative, it is possible that the large body sizes are related to achieving higher competitive strength during the period of high densities, when the water clarity is relatively high and thus food quantity low, while subsequent predation pressure by fish is so large that the population is eradicated irrespective of variation in body size.

We observed much variation between the two inbred families we studied in the degree of purging, the degree of inbreeding depression, and the capacity to show adaptive phenotypic plasticity. In this respect, we observed an interesting trade-off in the fact that the inbred family that suffered only mild initial purging (inbred family I2) not only suffered strong inbreeding depression for several life history traits, but also lacked the capacity to respond to the presence of fish kairomones by adaptive phenotypic plasticity. In contrast, inbred family I3 suffered strong mortality in the first 5–6 generations after hatching, but inbreeding depression for both life history and phenotypic plasticity in the remaining clones was mild or absent. Even though we studied only two inbred families, this is an interesting finding, worthy of further research. The absence of strong inbreeding depression for phenotypic plasticity in selfed offspring we find here provides some support for the hypothesis that phenotypic plasticity is mediated by “allelic sensitivity” (Via et al. 1995). Sensitivity of alleles to the environment is less dependent on dominance effects, as the presence of a single sensitive allele would already allow an individual to respond plastically to a range of environments. Phenotypic plasticity through “allelic sensitivity” might thus be less impacted by increased levels of homozygosity than phenotypic plasticity through “plasticity genes,” where an increase in homozygosity leads to a decreased diversity of plasticity genes, and thus decreased opportunities to express phenotypic plasticity. Alternatively, it is possible that purging of >50% of the lineages in inbred family I3 effectively removed a large proportion of genotypes that were highly homozygous for regulatory “plasticity genes” (and thus less capable of showing phenotypic plasticity), which would also explain the difference in inbreeding depression for phenotypic plasticity between the mildly purged inbred family I2 and the strongly purged inbred family I3. Third, a recent study by Van Buskirk and Steiner (2009) suggests that the costs of phenotypic plasticity are often not strong. If the energetic cost of plasticity is indeed low in *Daphnia*, it is not surprising that we found that the capacity for plasticity in one of two families we studied here was not affected at all by inbreeding. Overall, even though we quantified the consequences of inbreeding in only two inbred families, our results do clearly show that inbreeding depression varies strongly among families and that strong inbreeding can, but does not necessarily, impede adaptive phenotypic responses. The few other studies that jointly report inbreeding and plasticity were performed using *Drosophila* (Kristensen et al. 2011), frogs (Luquet et al. 2011), and freshwater snails (Auld and Relyea 2010), and these studies report both inbreeding depression for adaptive plasticity as well as no effects at all of inbreeding on adaptive plasticity. This study is the first study to quantify, albeit in two inbred families only, the effects of inbreeding on adaptive phenotypic plasticity. We show here that inbreeding depression for adaptive plasticity in *Daphnia* might be strong, but further research using a wide range of inbred families from different origins would definitely be worthwhile, as phenotypic plasticity is a very important life history strategy to deal with environmental circumstances in *Daphnia*, and inbreeding in natural *Daphnia* populations may occur commonly (see introduction).

### General conclusions

Our results suggest that there is a high variability among inbred families in the strength and expression of inbreeding depression, and in the degree to which the ability to show adaptive plasticity in response to predator stress is retained upon inbreeding. In one of the studied inbred families (I3), life history trait values and the ability to show adaptive phenotypic plasticity upon fish predation pressure approached the values of an outbred population after a phase of strong purging under benign conditions. In the other inbred family (I2), overall inbreeding depression for both life history and phenotypic plasticity was strong. While our results in general point to important fitness costs of inbreeding, in line with prior studies on inbreeding (Ebert et al. 2002; Keller and Waller 2002), they also show that purging from (sub)lethal alleles may, depending on the genotype of the mother, largely free inbred populations from their fitness cost. This may have important ramifications for *Daphnia* populations that are colonized by few individuals. If a population is founded by a single to a few individuals, the hatchlings of the subsequent growing season will exhibit strong inbreeding depression as they are the result of genetic selfing (as in this study). Ebert et al. (2002) showed that immigrant outbred genotypes rapidly overtake such a population. However, if no additional immigration occurs (because of an overall low dispersal rate) during the start of the growing season, initial purging may clear the inbred population largely from inbreeding depression. As half of the offspring clones are eliminated in this process, population growth of that population will initially be strongly reduced as compared to an outbred population, but this would hardly make a difference as long as no outbred lineages meanwhile colonized the habitat. Depending on the genotypic identity of the founder individual, the resulting inbred population may therefore perform similarly to an outbred population and may well have similar capacities to show adaptive phenotypic plasticity responses.
